# Insomnia severity and quality of life before and after comprehensive health resort treatment across BMI categories

**DOI:** 10.3389/fpubh.2026.1947274

**Published:** 2026-10-21

**Authors:** Grzegorz Onik, Kamil Barański, Krzysztof Suszyński

**Affiliations:** 1 Department of Physical Medicine, Faculty of Health Sciences in Katowice, Medical University of Silesia in Katowice, Katowice, Poland; 2 Department of Epidemiology, Faculty of Medical Sciences in Katowice, Medical University of Silesia in Katowice, Katowice, Poland; 3 Department of Sports Medicine and Physiology of Physical Effort, Faculty of Health Sciences in Katowice, Medical University of Silesia in Katowice, Katowice, Poland

**Keywords:** body mass index, health resort treatment, insomnia, obesity, quality of life

## Abstract

**Background:**

Excessive body weight is associated with numerous adverse health consequences, including insomnia and reduced quality of life. To date, the impact of body mass index on the effectiveness of comprehensive health resort treatment in terms of insomnia and quality of life has not been investigated. This study aimed to evaluate whether treatment-related improvements in insomnia and quality of life differ across BMI categories and whether BMI and weight loss are associated with treatment effectiveness.

**Methods:**

The study included 90 individuals (18 men and 72 women) with BMI ranging from 21.63 to 34.01 kg/m2 (mean: 27.82 ± 3.23 kg/m2) who underwent 21-day comprehensive health resort treatment. Participants were divided into three groups according to BMI category: normal weight, overweight, and class I obesity. Insomnia was assessed using the Athens Insomnia Scale (AIS), whereas health-related quality of life was assessed using the EQ-5D-5L questionnaire. Measurements were performed before and after completion of the 21-day treatment.

**Results:**

Changes in AIS scores (*p* = 0.23), EQ-5D-5L index values (*p* = 0.35), and EQ-5D-5L VAS scores (*p* = 0.88) following treatment did not differ significantly across BMI groups. BMI was not associated with changes in AIS scores (*β* = 0.28; *p* = 0.29), EQ-5D-5L index (*β* = −0.001; *p* = 0.67), or EQ-5D-5L VAS scores (*β* = 0.53; *p* = 0.33). Regression simulations indicated that hypothetical reductions in BMI (*β* = −0.380; *p* = 0.02) and body weight (*β* = −0.101; *p* = 0.04) would be associated with greater improvements in EQ-5D-5L VAS scores.

**Conclusion:**

Improvements in insomnia severity and quality of life were observed following comprehensive health resort treatment, with no statistically significant differences in these changes between BMI groups. A model-based simulation suggested that a hypothetical 3% weight loss might be associated with a small improvement in perceived health status. Further studies are needed to confirm these findings.

## 1 Introduction

Obesity is a major public health challenge, affecting approximately 1 billion people worldwide in 2022, with the number projected to reach 2 billion by 2035, resulting in a substantial economic and social burden (1). Currently, approximately 60% of adults in Europe are overweight, and nearly 25% are living with obesity (2). Obesity is associated with numerous adverse health consequences, including cardiovascular disease, respiratory disorders, musculoskeletal conditions, and an increased risk of several types of cancer (1, 2). In addition to its physical consequences, obesity is strongly associated with mental health disorders, including depression, anxiety (3), and sleep disturbances (4, 5). A recent meta-analysis showed that individuals with BMI ≥ 25 kg/m^2^ had 33% higher odds of sleep disturbances than those with normal body weight (6). Similarly, Kyrtsopoulos et al. (7) reported that class II obesity was associated with an increased risk of insomnia. Furthermore, excess body weight has been consistently linked to reduced quality of life (8–10). Given the well-established association of excess body weight with sleep disturbances and reduced quality of life, obesity represents an important clinical and public health challenge.

Different strategies have been implemented to improve sleep quality in individuals with obesity, including behavioral interventions, dietary interventions, physical activity and exercise, pharmacotherapy, and surgical procedures (11–13). Relatively little attention has been paid to the application of balneotherapy and comprehensive health resort treatment in individuals with overweight and obesity. Most studies investigating balneotherapy and health resort treatment in individuals with overweight and obesity have focused on body weight management (14–16) and selected functional outcomes (17). Even though health resort treatment has been associated with improvements in sleep quality (18–20), the impact of comprehensive health resort treatment on insomnia in individuals with overweight and obesity remains insufficiently investigated. To our knowledge, only one study has specifically evaluated sleep quality in women with morbid obesity. Koçak et al. (15) reported that a three-week balneotherapy program was associated with significant improvements in sleep quality. Moreover, recent studies have consistently demonstrated that balneotherapy and comprehensive health resort treatment are associated with improvements in quality of life across a wide range of medical conditions (21, 22). However, their effects in individuals with overweight and obesity have not been extensively investigated. Nevertheless, the available evidence suggests that comprehensive health resort treatment may improve quality of life in this population (15, 16, 23).

Considering this knowledge gap, we hypothesized that BMI may influence the effectiveness of comprehensive health resort treatment, resulting in differences in treatment-related improvements in insomnia symptoms and quality of life among individuals with normal weight, overweight, and obesity.

## 2 Materials and methods

### 2.1 Study group

This was a prospective, single-center, observational pre-post study in which insomnia severity and quality of life were assessed before and after a 21-day comprehensive health resort treatment program. The study was conducted between February and June 2025 at the Rehabilitation Hospital and Sanatorium in Goczałkowice-Zdrój, Poland. Situated in the Oświęcim Valley, Goczałkowice-Zdrój is classified as a lowland health resort and covers an area of 4,864 hectares. The town was officially granted health resort status in 2016 (24, 25). During the study period, 238 individuals initiated and completed the entire 21-day treatment program. All individuals were screened for eligibility. Of these, 154 met the age criterion, while 54 were excluded because they met one or more exclusion criteria. Consequently, 100 participants who met all eligibility criteria and completed both the baseline and post-treatment assessments were included in the initial study sample. The sample comprised 21 men and 79 women aged 40–79 years (mean age: 69.28 ± 9.29 years). Participants’ body mass index (BMI) ranged from 21.63 to 42.86 kg/m^2^ (mean: 28.77 ± 4.26 kg/m^2^). During the analytical stage, 10 individuals with a BMI ≥ 35 kg/m^2^ were excluded *post hoc* because their limited number precluded meaningful stratification into separate class II and class III obesity groups. Consequently, the final study cohort comprised 90 participants (18 men and 72 women) with a mean age of 69.32 ± 9.64 years. Figure 1 presents the participant selection process.

**Figure 1:**
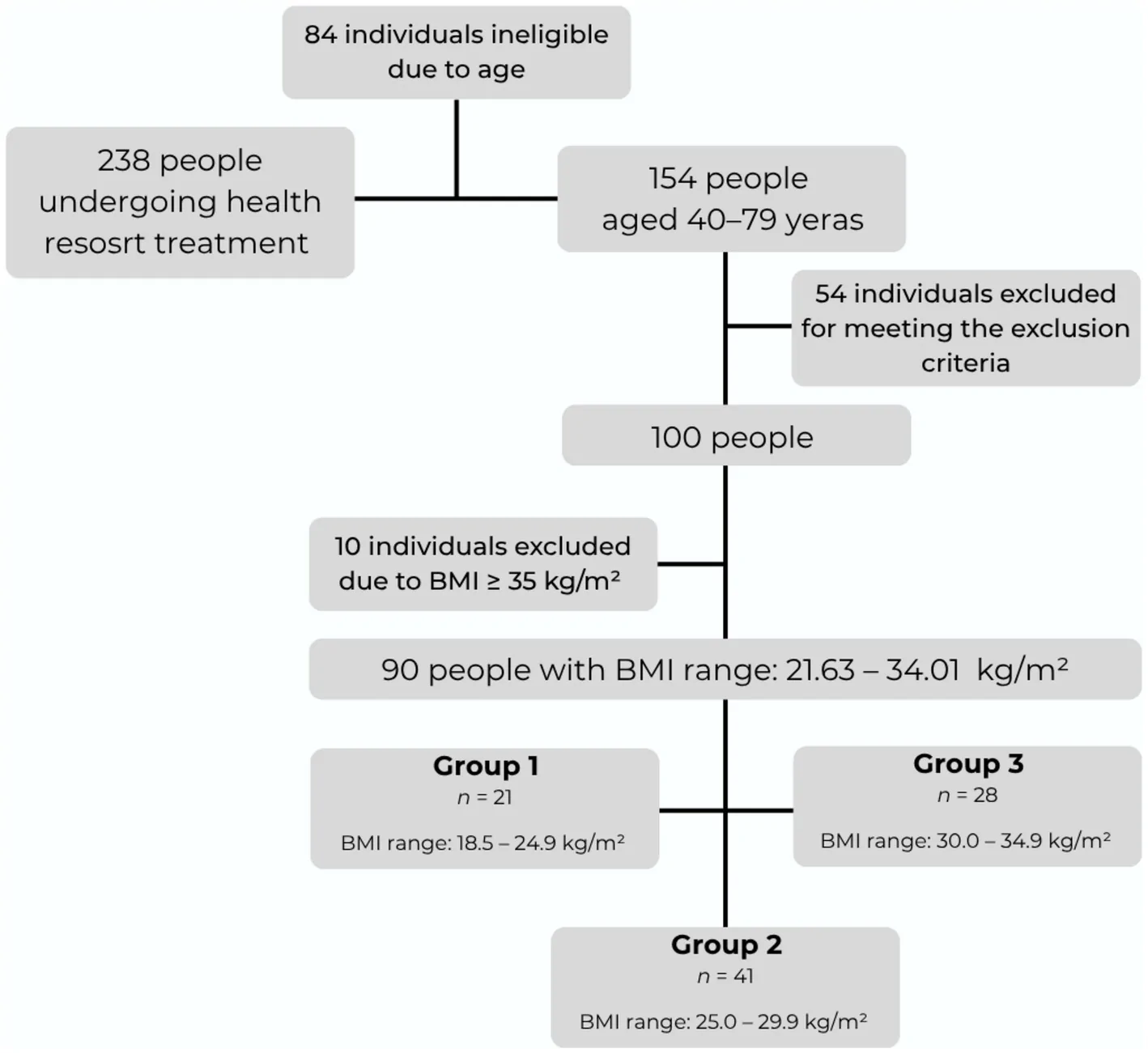
Participant selection flowchart.

Participants were divided into three groups according to BMI. Group 1 included individuals with a BMI of 18.5–24.9 kg/m^2^ and served as the reference BMI group. Group 2 comprised overweight individuals (BMI 25.0–29.9 kg/m^2^), whereas Group 3 included obese individuals with a BMI of 30.0–34.9 kg/m^2^ (class I obesity). Detailed participant characteristics are presented in Table 1.

**Table 1:** Baseline characteristics of participants stratified by BMI range.

Gender	Group 1 (*n* = 21)	Group 2 (*n* = 41)	Group 3 (*n* = 28)	*χ^2^*	*p* ^1^
Men (%)	9.52	24.39	21.43	1.97	0.37
Women (%)	90.48	75.61	78.57		

Anthropometric variable	Group 1 (*n* = 21)	Group 2 (*n* = 41)	Group 3 (*n* = 28)	*F*	*p* ^2^	Post-hoc test Bonferroni
Age [years]	70.67 ± 7.17	68.78 ± 10.83	69.11 ± 9.26	0.27	0.76	–
Height [m]	1.65 ± 0.08	1.65 ± 0.09	1.65 ± 0.08	0.08	0.93	–
Weight [kg]	63.86 ± 7.35	74.63 ± 8.70	86.46 ± 9.47	41.56	< 0.0001	Group 1 vs. Group 2: *p* < 0.0001 Group 1 vs. Group 3: *p* < 0.0001 Group 2 vs. Group 3: *p* < 0.0001
BMI [kg/m^2^]	23.45 ± 1.06	27.52 ± 1.38	31.55 ± 1.14	258.27	< 0.0001	Group 1 vs. Group 2: *p* < 0.0001 Group 1 vs. Group 3: *p* < 0.0001 Group 2 vs. Group 3: *p* < 0.0001
SBP [mmHg]	140.24 ± 8.01	139.68 ± 7.17	143.32 ± 6.89	2.20	0.12	–
DBP [mmHg]	74.67 ± 5.90	77.12 ± 5.09	74.06 ± 8.57	7.26	0.001	Group 1 vs. Group 2: *p* = 0.25 Group 1 vs. Group 3: *p* = 0.001 Group 2 vs. Group 3: *p* = 0.05
HR [beats/min]	75.24 ± 7.24	73.10 ± 9.50	74.04 ± 8.60	0.42	0.66	–

Categorical variables are shown as percentages; continuous variables are reported as mean ± SD. *p*^1^ and *p*^2^, *p* value; *χ*^2^, chi-square statistic; *F*, F-statistic.

In the study group, the most frequent comorbidity was hypertension, observed in 53.33% of participants, with no significant differences between BMI groups. Hypothyroidism was the second most common condition, affecting 23.33% of participants. Diabetes mellitus was present in 13.33% of participants, with a mean baseline glucose level of 115.79 ± 17.45 mg/dL, and its frequency did not differ significantly between groups. One-way ANOVA showed no significant differences in glucose levels between BMI groups [*F* (2,11) = 1.87, *p* = 0.20]. The frequency of peripheral artery disease differed significantly between BMI groups (*χ*^2^ = 6.72; *p* = 0.04), with cases observed only in Group 1 (9.52%). Table 2 summarizes the frequency of comorbidities across BMI groups.

**Table 2:** Frequency of comorbidities stratified by BMI range.

Comorbidity	Group 1	Group 2	Group 3	*χ* ^2^	*p*
Hypertension	47.62%	53.66%	57.14%	0.44	0.80
Ischemic heart disease	14.29%	7.32%	10.71%	0.77	0.68
Peripheral artery disease	9.52%	–	–	6.72	0.04
Mitral regurgitation	4.76%	–	7.14%	2.81	0.25
Varicose veins	4.76%	14.63%	28.57%	5.12	0.07
Diabetes mellitus	9.52%	9.76%	21.43%	2.31	0.32
Asthma	-	4.88%	3.57%	1.03	0.60
Pneumoconiosis	-	2.44%	–	1.21	0.55
Hyperthyroidism	-	4.88%	3.57%	1.03	0.60
Hypothyroidism	14.29%	21.95%	32.14%	2.22	0.33
Hyperuricemia	–	9.76%	10.71%	2.33	0.31
Gout	–	4.88%	–	2.45	0.30
Chronic kidney disease	–	–	3.57%	2.24	0.33
Benign prostatic hyperplasia	–	4.88%	3.57%	1.03	0.60
Psoriasis	–	–	3.57%	2.24	0.33
Psoriatic arthritis	–	–	3.57%	2.24	0.33
Rheumatoid arthritis	–	2.44%	3.57%	0.72	0.70
Osteoporosis	9.52%	9.76%	7.14%	0.15	0.76
Spondyloarthritis	–	–	3.57%	2.24	0.33
Gastroesophageal reflux disease	4.76%	4.88%	–	1.40	0.50
Depression	–	7.32%	–	3.71	0.16

Values are expressed as percentages. *χ*^2^, chi-square statistic; *p*, *p*-value.

### 2.2 Inclusion and exclusion criteria

Participants between 40 and 79 years of age who underwent and completed a 21-day program of comprehensive health resort treatment were considered for inclusion. Subjects were not eligible if they presented with significant comorbid conditions, including cardiovascular diseases (heart failure exceeding NYHA class II, previous myocardial infarction, history of coronary artery bypass grafting, prior percutaneous coronary intervention with stent placement, or implanted pacemaker), neurological disorders (including Parkinson’s disease, multiple sclerosis, motor deficits such as paresis or spasticity, stroke, meningitis, or cognitive impairment), chronic obstructive pulmonary disease, a history of limb amputation, or active or past malignancy.

### 2.3 Treatment course

Comprehensive sanatorium treatment lasted 21 days. The regimen was personalized based on a pre-treatment clinical assessment. The therapeutic program was conducted 6 days a week and comprised no fewer than three procedures daily, in compliance with statutory requirements governing spa rehabilitation. Balneological treatments constituted an integral component of the program, with each treatment day including at least one procedure based on natural therapeutic resources (brine-based or peloid-based therapies), combined with additional physiotherapy interventions. Peloid therapy was the most frequently applied balneological intervention, administered to 95.56% of participants at 40 °C for 15 min. Brine therapy was the second most common, used in 75.56% of participants at 38–40 °C for 15 min. Vacuum massage was performed in 53.33% of participants and was applied significantly more often in overweight individuals (Group 2; *χ*^2^ = 7.60, *p* = 0.02). LED therapy was the least frequently applied intervention, occurring in only 2.22% of participants. The frequency of other interventions did not differ between BMI groups. Frequencies of physical medicine and balneological interventions are summarized in Table 3. Physical therapy modalities and balneological treatments were combined with guided exercise sessions aimed at improving joint mobility, muscular endurance, strength, coordination, and balance. All participants attended physiotherapist-supervised exercise sessions. Participants also attended health education sessions covering nutrition, regular physical activity, and the prevention of tobacco, alcohol, and other substance use.

**Table 3:** Use of physical medicine and balneotherapy across BMI groups.

Modality	Group 1	Group 2	Group 3	*χ* ^2^	*p*
Vacuum massage	33.33%	68.29%	46.43	7.60	0.02
Classical massage	28.57%	7.32%	17.86%	4.94	0.08
Mechanical massage	38.10%	12.20%	25%	5.57	0.06
Pearl bath	14.29%	29.27%	14.29%	3.00	0.22
Peloids	95.24%	95.12%	96.43	0.07	0.96
Brine	85.71%	73.17%	71.43%	1.56	0.50
Local cryotherapy	9.52%	12.20%	25.00%	2.83	0.24
Whirlpool bath	9.52%	19.51%	28.57%	2.73	0.26
Infrared light	4.76%	7.32%	10.71%	0.61	0.74
Magnetledtherapy	4.76%	7.32%	–	2.10	0.35
TENS	9.52%	17.07%	7.14	1.71	0.42
Low-level laser therapy	23.81%	14.63%	25%	1.37	0.51
Ultrasounds	14.29%	2.44%	7.14%	3.15	0.21
Iontophoresis	4.76%	7.32%	7.14%	0.16	0.92
LED therapy	4.76%	–	3.57%	1.79	0.41

Values are expressed as percentages. *χ*^2^, chi-square statistic; *p*, *p*-value.

### 2.4 Methods of evaluation

Each participant underwent routine clinical examinations before and after treatment. During these pre- and post-treatment assessments, insomnia severity and quality of life were evaluated, resulting in two measurements. Insomnia severity was measured using the Athens Insomnia Scale (AIS), while quality of life was assessed with the European Quality of Life Five Dimensions – Five Levels (EQ-5D-5L). The AIS is a standardized tool composed of eight questions evaluating sleep quality across several dimensions, including difficulty in initiating and maintaining sleep, overall sleep quality and duration, and daytime functioning. The Athens Insomnia Scale yields a total score between 0 and 24, with higher values indicating more severe insomnia (26–28). A threshold of 6 points has been recommended to identify insomnia with high sensitivity and specificity (29). The scale has been validated for application in the Polish population (30).

Health-related quality of life was assessed with the European Quality of Life Five Dimensions – Five Levels questionnaire (EQ-5D-5L), a standardized tool consisting of five domains: mobility, self-care, usual activities, pain/discomfort, and anxiety/depression (31). Participants’ overall perceived health was recorded using the EuroQol Visual Analog Scale (EQ-VAS), where higher values indicate better self-rated health (32). The EQ-5D-5L index scores were computed following the methodology described by Golicki et al. (33, 34). This use of the EQ-5D-5L was officially registered with EuroQol under number 70086.

Individuals gave consent agreement for the proposed treatment protocol and related pre- and post-treatment examinations. Ethical approval was waived by the Bioethical Committee of the Medical University of Silesia in Katowice due to the non-experimental nature of the study (Decision No.: BNW/NWN/0052/KB/41/25, 4 February 2025).

### 2.5 Study outcomes

The primary outcome was the comparison of treatment-related changes in insomnia severity and quality of life, as measured by the Athens Insomnia Scale (AIS) and the European Quality of Life Five Dimensions–Five Levels (EQ-5D-5L), across BMI groups following comprehensive health resort treatment. Secondary outcomes included the assessment of within-group changes in AIS and EQ-5D-5L scores and the evaluation of whether BMI and weight loss during treatment were associated with treatment effectiveness.

### 2.6 Statistical analysis

The distribution of continuous variables was examined using the Shapiro–Wilk test. Continuous variables are presented as means ± standard deviation (SD), whereas categorical variables are reported as numbers and percentages. Homogeneity of variance was assessed with Bartlett’s test when analysis of variance was applied. Baseline characteristics were compared across the three BMI groups: normal weight, overweight, and class I obesity. Continuous variables meeting the assumption of normality were compared using one-way analysis of variance. When appropriate, pairwise comparisons were performed with Bonferroni adjustment for multiple testing. Variables that did not meet the assumption of normality were compared using the Kruskal–Wallis test. Categorical variables were analyzed using the chi-square test.

Changes between baseline and post-treatment measurements were assessed separately within each BMI group. For normally distributed continuous outcomes, paired Student’s t-tests were used. For non-normally distributed or ordinal variables, comparisons were performed using the Wilcoxon signed-rank test. Changes in Athens Insomnia Scale score (ΔAIS), EQ-5D-5L index (ΔEQ-5D-5L index), and EQ-5D-5L VAS score (ΔEQ-5D-5L VAS) were calculated as post-treatment minus baseline values. Accordingly, improvement was reflected by negative ΔAIS values and positive ΔEQ-5D-5L index and ΔEQ-5D-5L VAS values. Differences in treatment response between the baseline BMI categories were first assessed by comparing ΔAIS, ΔEQ-5D-5L index, and ΔEQ-5D-5L VAS across the three groups. Within-group changes in AIS and EQ-5D-5L VAS were reported as raw mean changes with 95% confidence intervals and standardized using Cohen’s (d_z_) with corresponding 95% confidence intervals. One-way analysis of variance with Bonferroni-adjusted pairwise comparisons was used for normally distributed change scores. The overall magnitude of between-group differences was quantified using eta-squared (η^2^) with a 95% confidence interval, and pairwise mean differences were reported with Bonferroni-adjusted 95% confidence intervals. The Kruskal–Wallis test was used for non-normally distributed change scores. For the EQ-5D-5L index, epsilon-squared (ε^2^) and bootstrap 95% confidence intervals were reported.

Multivariable linear regression models were then fitted to determine whether treatment response differed according to baseline BMI category after adjustment for potential confounding factors. Separate models were constructed for ΔAIS, ΔEQ-5D-5L index, and ΔEQ-5D-5L VAS. Participants with normal weight were treated as the reference category, and the treatment response observed in participants with overweight and class I obesity was compared with that in the reference group. The models were adjusted for age, sex, systolic and diastolic blood pressure, heart rate, and the presence of any comorbidity. Comorbidity status was included as a single binary covariate (0 = no reported comorbidities; 1 = presence of at least one reported comorbidity), rather than by entering individual medical conditions as separate covariates. Each multivariable model included eight predictor parameters, excluding the intercept. The models for ΔAIS, ΔEQ-5D-5L index, and ΔEQ-5D-5L VAS included 89, 85, and 90 participants, respectively, corresponding to approximately 11.1, 10.6, and 11.3 observations per predictor parameter. Thus, all models met the conventional heuristic of at least 10 observations per estimated predictor parameter. Covariates were selected *a priori* based on their potential clinical relationships with BMI, insomnia, and quality of life rather than on statistical significance in univariable analyses. No automated variable-selection procedure was used. In additional analyses, BMI and body weight were examined as continuous predictors. Separate regression models were used to assess whether higher baseline BMI or body weight was associated with the magnitude of change in each study outcome. BMI category and continuous BMI were not entered into the same regression model. Regression coefficients, 95% confidence intervals, and *p*-values were reported for individual predictors. Overall model significance was assessed using the F test, and the proportion of variance explained by each model was expressed using the coefficient of determination (R^2^).

Multivariable logistic regression models were fitted to examine the associations between individual therapeutic modalities and post-treatment insomnia status (defined as an AIS score ≥6) as well as improvement in quality of life (EQ-5D-5L index and EQ-5D-5L VAS). Improvement in quality of life was treated as a dichotomous outcome (1 = improvement, 0 = no improvement), based on changes (Δ) in the EQ-5D-5L index and EQ-5D-5L VAS scores. To ensure model stability and minimize sparse-data bias, only modalities administered to at least 10% of participants were included in the analyses. All models were adjusted for BMI group.

Regression-based estimates were also used to explore the expected changes in ΔAIS, ΔEQ-5D-5L index, and ΔEQ-5D-5L VAS associated with hypothetical reductions in body weight and BMI. Reductions from 0.5 to 3.0%, in increments of 0.5 percentage points, were considered. These estimates were derived from the coefficients obtained from models in which body weight or BMI was entered as a continuous predictor. Multicollinearity was assessed using variance inflation factors and tolerance statistics. A variance inflation factor greater than 5 or a tolerance value below 0.20 was considered indicative of potentially problematic multicollinearity. No problematic multicollinearity was identified in the multivariable models.

Statistical analyses were performed using Stata 19 (StataCorp LLC, College Station, TX, United States). No *a priori* sample-size calculation was performed. The study included all eligible participants treated during the predefined study period. Given the fixed sample size, the precision of the estimates was evaluated using effect sizes and their corresponding 95% confidence intervals. All statistical tests were two-sided, and a *p*-value below 0.05 was considered statistically significant.

## 3 Results

During the baseline measurement, 45.56% of participants met the criteria for insomnia based on the Athens Insomnia Scale cut-off value of six points. In Group 1, 38.10% of participants scored above this threshold. Among overweight individuals (Group 2), 43.90% met the criteria for insomnia, while in obese participants (Group 3), 53.57% were diagnosed with insomnia upon admission. Nevertheless, statistical analysis did not reveal a significant difference in insomnia frequency between the groups (*χ*^2^ = 1.24; *p* = 0.54). Despite the fact that, during the pre-treatment evaluation, the mean Athens Insomnia Scale score was approximately 25% higher in overweight participants (Group 2) and approximately 36% higher in obese participants (Group 3) compared with participants in Group 1, statistical analysis did not reveal significant differences between the groups [*F* (2, 86) = 1.12; *p* = 0.33]. Bonferroni post-hoc tests showed no significant pairwise differences, and Bartlett’s test confirmed equal variances [χ^2^ (2) = 0.27; *p* = 0.88].

Following comprehensive health resort treatment, the prevalence of insomnia among participants, assessed using the Athens Insomnia Scale, decreased to 37.78% (McNemar’s *χ*^2^ (1) = 5.44, *p* = 0.02). In individuals with a normal BMI range, the frequency of insomnia during the post-treatment evaluation was 33.33% [McNemar’s *χ*^2^ (1) = 1.00; *p* = 0.32]. In Group 2, insomnia frequency decreased to 31.71% [McNemar’s *χ*^2^ (1) = 5.00; *p* = 0.03], while in Group 3 it decreased to 50% [McNemar’s *χ*^2^ (1) = 0.33; *p* = 0.56]. Statistical analysis did not reveal significant differences in insomnia frequency between the groups (*χ*^2^ = 2.60; *p* = 0.28). Following sanatorium treatment, insomnia severity was approximately 7% lower in Group 2 and approximately 39% lower in Group 3 compared with individuals with a normal BMI range (Group 1). Statistical analysis did not reveal significant differences between the groups [*F* (2, 86) = 1.81; *p* = 0.17]. Bonferroni post-hoc tests showed no significant pairwise differences, and Bartlett’s test confirmed equal variances [*χ*^2^ (2) = 1.62; *p* = 0.45]. Figure 2 presents Athens Insomnia Scale (AIS) scores during pre- and post-treatment evaluations in groups stratified by BMI range.

**Figure 2:**
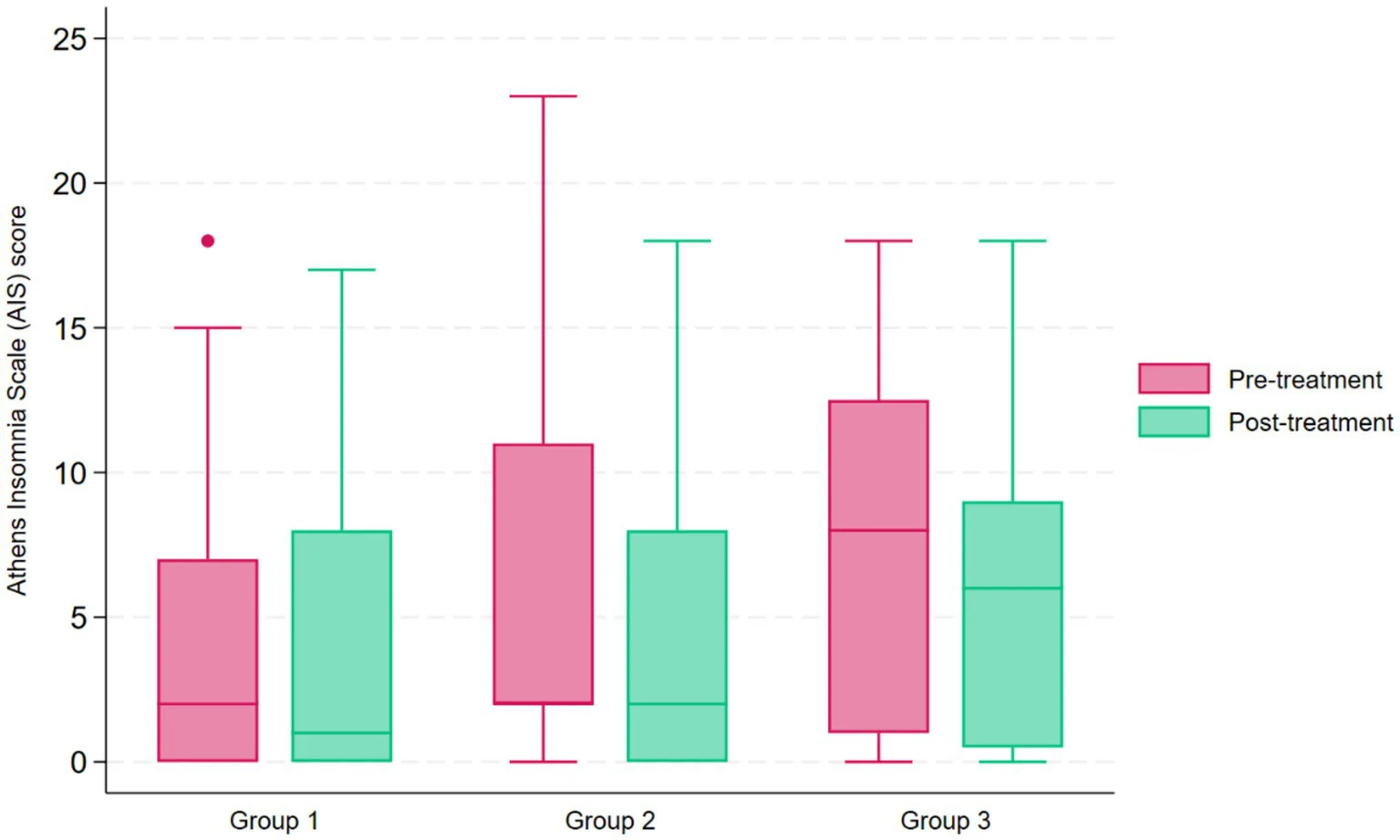
Athens insomnia scale (AIS) scores measured before and after treatment in participants stratified by BMI category.

Comprehensive health resort treatment resulted in a significant improvement in insomnia severity, as evaluated with the AIS, in all groups. In Group 1, insomnia severity decreased by approximately 23% (mean difference: 1.10 ± 2.27; *t* = 2.17; *p* = 0.04; 95% CI: 0.04 to 2.16). The corresponding standardized effect was moderate [(d_z_ = 0.48); 95% CI: 0.01 to 0.94]. A greater reduction of about 38% was observed in Group 2 (mean difference: 2.37 ± 3.01; *t* = 5.04; *p* < 0.0001; 95% CI: 1.42 to 3.31), corresponding to a moderate-to-large standardized effect (d_z_ = 0.79); 95% CI: 0.43 to 1.14), while in Group 3 the decrease was approximately 20% (mean difference: 1.50 ± 3.12; *t* = 2.54; *p* = 0.02; 95% CI: 0.29 to 2.71). The standardized effect in Group 3 was moderate (d_z_ = 0.48); 95% CI: 0.09 to 0.87). The largest mean reduction in AIS score was observed in Group 2. However, the overall magnitude of the between-group effect was small (*η*^2^ = 0.034; 95% CI: 0.000 to 0.121; *F*(2, 86) = 1.51; *p* = 0.23). In Bonferroni-adjusted pairwise comparisons, the mean AIS reduction was 1.27 points greater in Group 2 than in Group 1 (95% CI: −0.66 to 3.20), 0.40 points greater in Group 3 than in Group 1 (95% CI: −1.67 to 2.47), and 0.87 points greater in Group 2 than in Group 3 (95% CI: −0.87 to 2.61). All confidence intervals included zero. Bonferroni post-hoc tests showed no significant pairwise differences, and Bartlett’s test confirmed equal variances [*χ*^2^ (2) = 2.33; *p* = 0.31]. Figure 3 presents the mean change in Athens Insomnia Scale scores (ΔAIS) across the groups.

**Figure 3:**
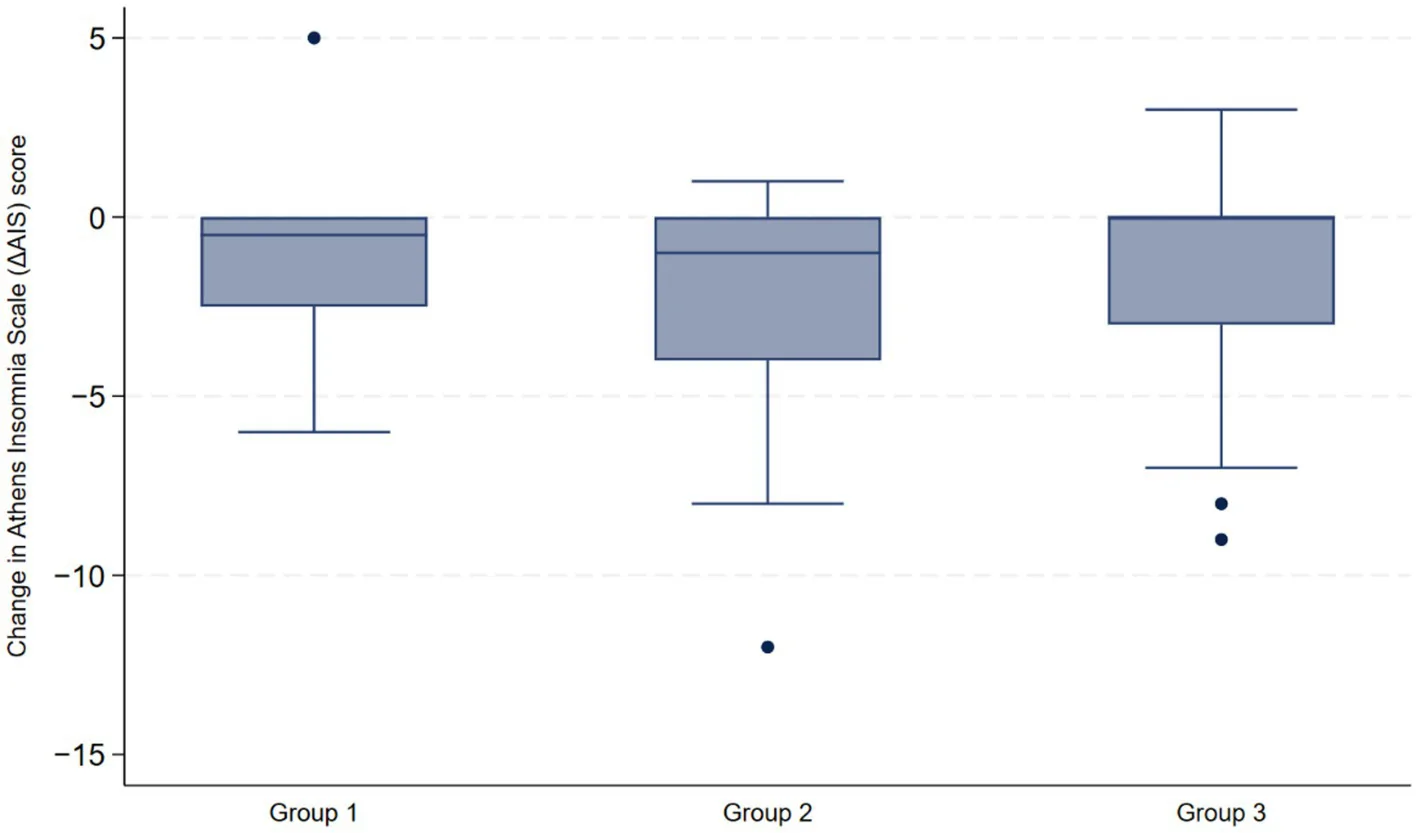
Changes in athens insomnia scale scores (ΔAIS) across BMI categories.

Complex health resort treatment resulted in a significant improvement in pain across all groups of participants. The remaining dimensions of the EQ-5D-5L remained unchanged following the intervention. No statistically significant differences between the patient groups were observed at either stage of the study. Table 4 presents detailed results of the EQ-5D-5L assessment.

**Table 4:** Changes in EQ-5D-5L dimensions between baseline and post-treatment across BMI groups.

Group 1	Mobility		Self-care		Usual activities		Pain/discomfort		Anxiety/depression	
	Before	After	Before	After	Before	After	Before	After	Before	After
No problems	75%	80%	100%	95%	90%	95%	40%	65%	90%	100%
Slight problems	25%	20%	–	5%	10%	5%	55%	35%	10%	–
Moderate problems	–	–	–	–	–	–	5%	–	–	–
Severe problems	–	–	–	–	–	–	–	–	–	–
Extreme problems/ unable to do	–	–	–	–	–	–	–	–	–	–
z	0.58		−1.00		0.58		2.49		1.41	
*p^1^*	0.56		0.32		0.56		0.01		0.16	

Group 2	Mobility		Self-care		Usual activities		Pain/discomfort		Anxiety/depression	
	Before	After	Before	After	Before	After	Before	After	Before	After
No problems	81.58%	78.95%	92.11%	92.11%	92.11%	89.47%	47.37%	60.53%	97.37%	97.37%
Slight problems	5.26%	13.16%	5.26%	7.89%	5.26%	10.53%	28.95%	39.47%	2.63%	2.63%
Moderate problems	13.16%	7.89%	2.63%	-	2.63%	-	23.68%	–	–	–
Severe problems	–	–	–	–	–	–	–	–	–	–
Extreme problems/ unable to do	–	–	–	–	–	–	–	–	–	–
z	0.58		1.00		0.00		3.74		0.00	
*p^1^*	0.56		0.32		1.00		0.0002		1.00	

Group 3	Mobility		Self-care		Usual activities		Pain/discomfort		Anxiety/depression	
	Before	After	Before	After	Before	After	Before	After	Before	After
No problems	70.37%	77.78%	85.19%	85.19%	88.89%	92.59%	40.74%	48.15%	92.59%	85.19%
Slight problems	18.52%	14.81%	14.81%	14.81%	11.11%	7.41%	44.44%	48.15%	7.41%	14.81%
Moderate problems	7.41%	3.70%	–	–	–	–	11.11%	3.70%	–	–
Severe problems	3.70%	3.70%	–	–	–	–	3.70%	–	–	–
Extreme problems/ unable to do	–	–	–	–	–	–	–	–	–	–
z	1.41		–		1.00		2.23		−1.41	
*p^1^*	0.16		1.00		0.32		0.03		0.16	

Groups comparison	Mobility		Self-care		Usual activities		Pain/discomfort		Anxiety/depression	
	Before	After	Before	After	Before	After	Before	After	Before	After
χ*^2^*	0.87	0.10	3.22	1.46	0.16	0.56	0.13	1.84	1.43	5.80
*p^2^*	0.65	095	0.20	0.48	0.92	0.76	0.94	0.40	0.49	0.06

Values are expressed as percentages. z, Wilcoxon test statistic; *χ*^2^, Kruskal–Wallis test statistic; *p*^1^, *p*-value for intragroup comparisons before vs. after treatment; *p*^2^, *p*-value for intergroup comparisons between groups.

During the pre-treatment evaluation, the lowest EQ-5D-5L VAS scores were observed in obese participants (Group 3). However, statistical analysis revealed no significant differences between the groups [*F* (2, 87) = 0.63; *p* = 0.23]. Bonferroni *post hoc* tests indicated no significant pairwise differences, and Bartlett’s test confirmed the homogeneity of variances [χ^2^ (2) = 0.44; *p* = 0.80]. Following the completion of the comprehensive treatment, the highest EQ-5D-5L VAS scores were recorded in individuals with a normal BMI (Group 1). However, these differences did not reach statistical significance [*F* (2, 87) = 1.62; *p* = 0.20]. Consistently, Bonferroni *post hoc* tests showed no significant pairwise differences, while Bartlett’s test confirmed the homogeneity of variances [χ^2^ (2) = 0.63; *p* = 0.73].

EQ-5D-5L VAS scores increased between admission and discharge in all three BMI groups. In Group 1, the mean increase was 13.10 points (SD: 6.80; 95% CI: 10.00 to 16.18), corresponding to a large standardized effect (d_z_ = 1.93; 95% CI: 1.19 to 2.65). In Group 2, the mean increase was 12.20 points (SD: 6.33; 95% CI: 10.20 to 14.19), corresponding to a large standardized effect (d_z_ = 1.93; 95% CI: 1.40 to 2.44). In Group 3, the mean increase was 12.50 points (SD: 7.14; 95% CI: 9.73 to 15.27), corresponding to a large standardized effect (d_z_ = 1.75; 95% CI: 1.15 to 2.34). The overall between-group effect was very small [*η*^2^ = 0.003; 95% CI: 0.000 to 0.036; *F* (2, 87) = 0.13; *p* = 0.88]. Bartlett’s test supported the assumption of homogeneity of variances [*χ*^2^ (2) = 0.47; *p* = 0.79]. Figure 4 illustrates EQ-5D-5L VAS scores across the participant groups at pre- and post-treatment assessments.

**Figure 4:**
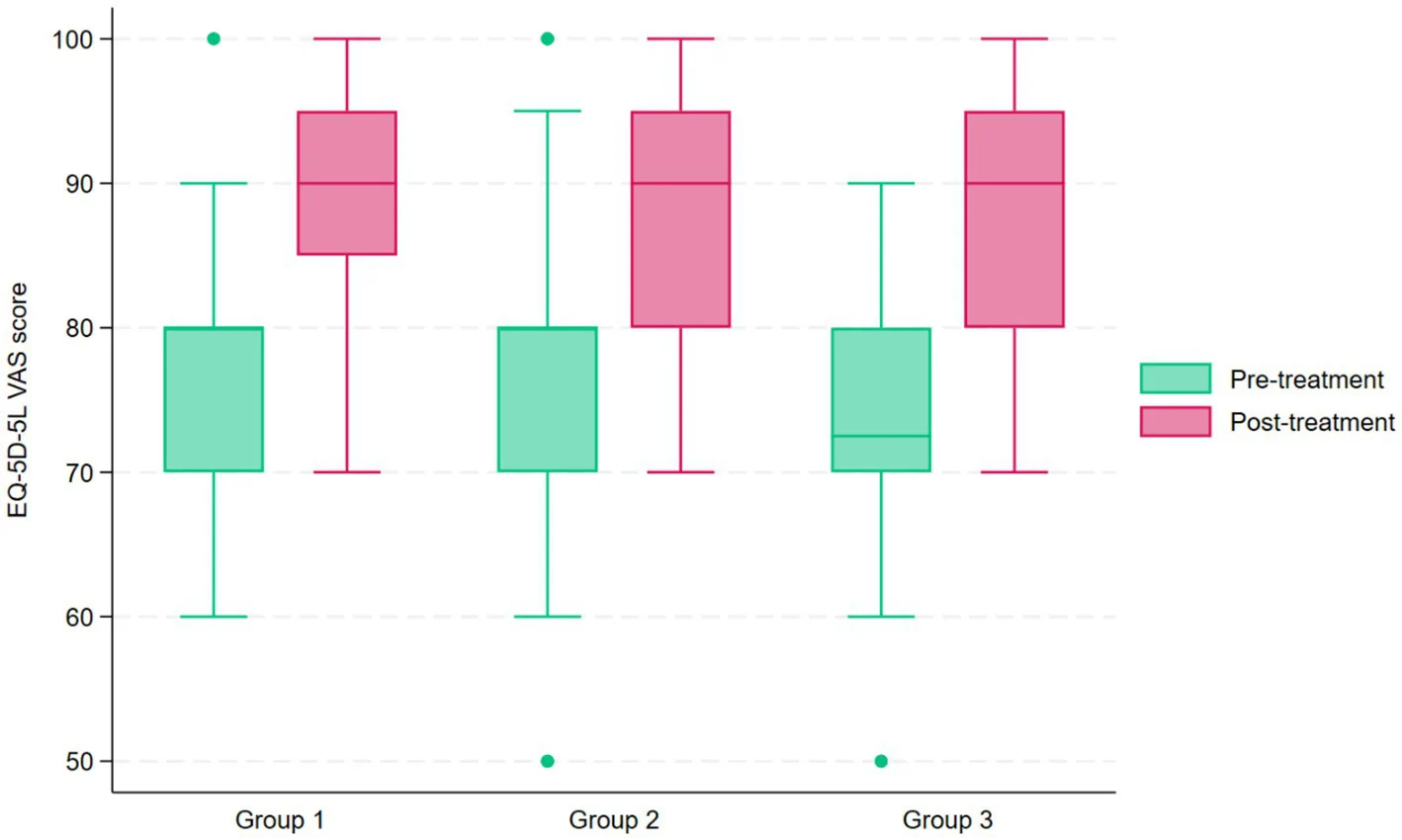
EQ-5D-5L VAS scores across BMI groups at pre- and post-treatment assessments.

In Bonferroni-adjusted pairwise comparisons, the mean EQ-5D-5L VAS improvement was 0.90 points lower in Group 2 than in Group 1 (95% CI: −5.29 to 3.49), 0.60 points lower in Group 3 than in Group 1 (95% CI: −5.32 to 4.12), and 0.30 points greater in Group 3 than in Group 2 (95% CI: −3.71 to 4.31). All confidence intervals included zero. Figure 5 presents the mean change in EQ-5D-5L VAS (ΔEQ-5D-5L VAS) across the groups.

**Figure 5:**
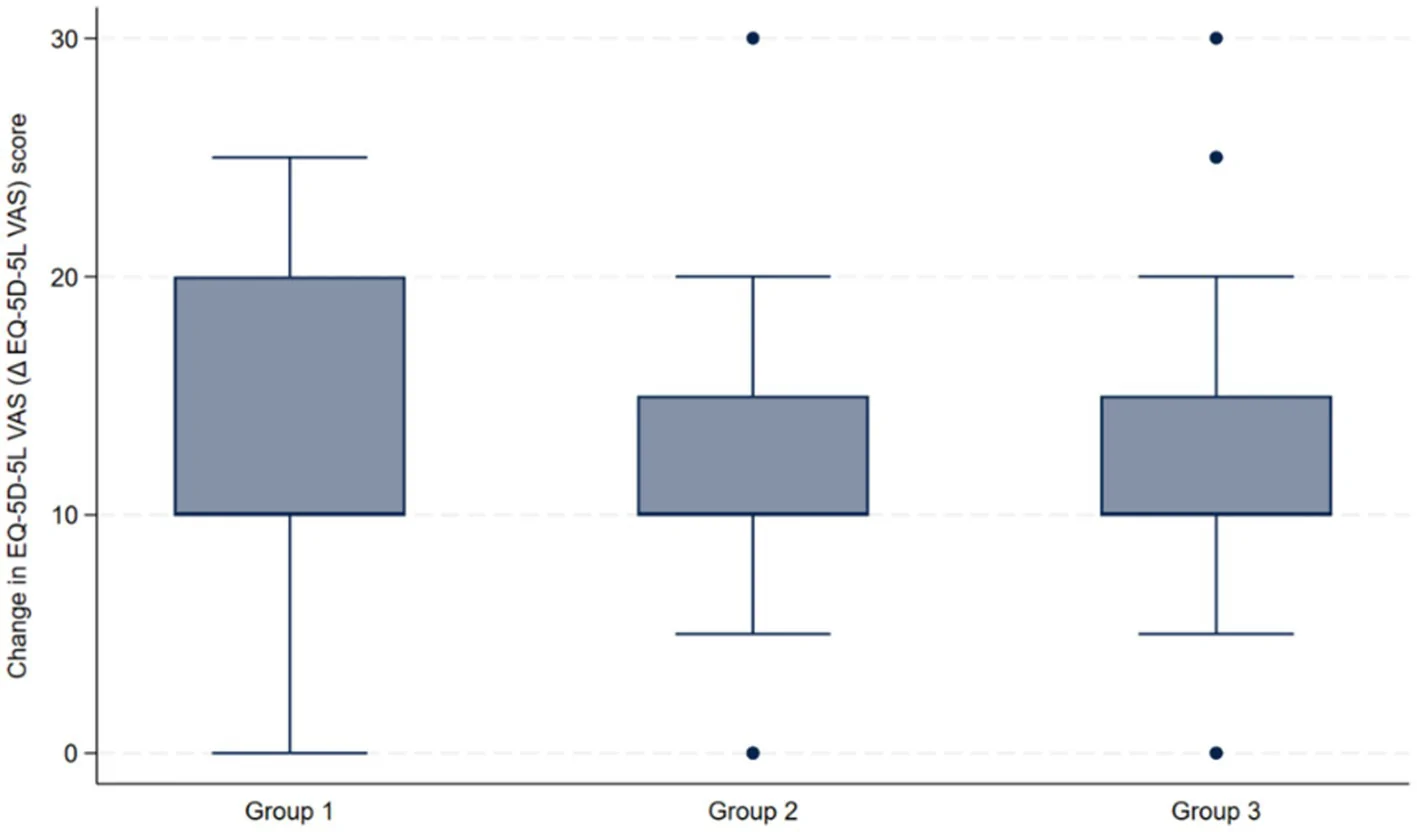
Changes in EQ-5D-5L VAS (ΔEQ-5D-5L VAS) across the study groups.

During pre- and post-treatment evaluations, the EQ-5D-5L index did not differ significantly between BMI groups. However, the highest values were observed in participants within the normal BMI range. Overall, the EQ-5D-5L index improved in 32.94% of individuals. In Group 1, improvement was noted in 40.00% of participants, in Group 2 in 36.84%, and in Group 3 in 22.22% of participants. The frequency of improvement did not differ significantly between the groups (*χ*^2^ = 2.12; *p* = 0.35). In Group 1, the mean EQ-5D-5L index increased by 0.011 (SD: 0.021; 95% CI: 0.001 to 0.021), corresponding to a moderate standardized effect (d_z_ = 0.54; 95% CI: 0.06 to 1.00). In Group 2, the mean increase was 0.009 (SD: 0.019; 95% CI: 0.002 to 0.015), corresponding to a small-to-moderate effect (d_z_ = 0.45; 95% CI: 0.11 to 0.78). In Group 3, the mean increase was 0.014 (SD: 0.047; 95% CI: −0.005 to 0.033), corresponding to a small effect (d_z_ = 0.30; 95% CI: −0.09 to 0.68). The overall between-group effect was small [η^2^ = 0.005; 95% CI: 0.000 to 0.051; *F* (2, 82) = 0.22; *p* = 0.80]. Consistently, the Kruskal–Wallis test did not demonstrate significant differences in EQ-5D-5L index changes between the BMI groups [*χ*^2^ (2) = 1.48; *p* = 0.48]. In Bonferroni-adjusted pairwise comparisons, the mean improvement was 0.002 lower in Group 2 than in Group 1 (95% CI: −0.023 to 0.019), 0.003 greater in Group 3 than in Group 1 (95% CI: −0.020 to 0.025), and 0.005 greater in Group 3 than in Group 2 (95% CI: −0.014 to 0.024). All confidence intervals included zero. Table 5 summarizes the EQ-5D-5L index values at admission and discharge together with the corresponding changes (ΔEQ-5D-5L index) across the study groups.

**Table 5:** EQ-5D-5L index values at admission and discharge and changes across BMI groups.

EQ-5D-5L index	Group 1 (*n* = 21)	Group 2 (*n* = 41)	Group 3 (*n* = 28)	*χ* ^2^	*p* ^1^
Pre-treatment	0.971 ± 0.027	0.968 ± 0.045	0.951 ± 0.082	0.81	0.67
Post-treatment	0.982 ± 0.024	0.977 ± 0.034	0.965 ± 0.045	1.69	0.43
*z*	−2.4	−2.87	−1.59	–	–
*p* ^2^	0.02	0.003	0.08	–	–
ΔEQ-5D-5L index	0.011 ± 0.021	0.009 ± 0.020	0.014 ± 0.047	2.08	0.35

Variables are reported as mean ± SD. *z*, Wilcoxon test statistic; *χ*^2^, Kruskal–Wallis test statistic; *p*^1^, *p*-value for intergroup comparisons between groups; *p*^2^, *p*-value for intragroup comparisons before vs. after treatment.

Multivariable regression models assessing the association between changes in Athens Insomnia Scale and body mass index adjusted for sex, age, blood pressure, heart rate, presence of comorbidities and study group were not statistically significant [*F* (8, 80) = 0.47; *p* = 0.87; *R*^2^ = 0.05]. Smilarly, changes in EQ-5D-5L index were not associated with body mass index in the studied individuals [*F* (8, 76) = 0.36; *p* = 0.94, *R*^2^ = 0.04]. This model was adjusted for sex, age, blood pressure, heart rate, comorbidities and study group. Although the multivariable regression model assessing changes in EQ-5D-5L VAS was statistically significant [*F* (8, 81) = 2.41; *p* = 0.02; *R*^2^ = 0.19], body mass index was not independently associated with changes in EQ-5D-5L VAS. The only significant predictor identified in the model was diastolic blood pressure (*β* = −0.32; *p* = 0.03; 95% CI: −0.60 to −0.04). All analyses were adjusted for sex, age, blood pressure, heart rate, comorbidities, and study group. The results of the multivariable regression analyses are presented in Figure 6.

**Figure 6:**
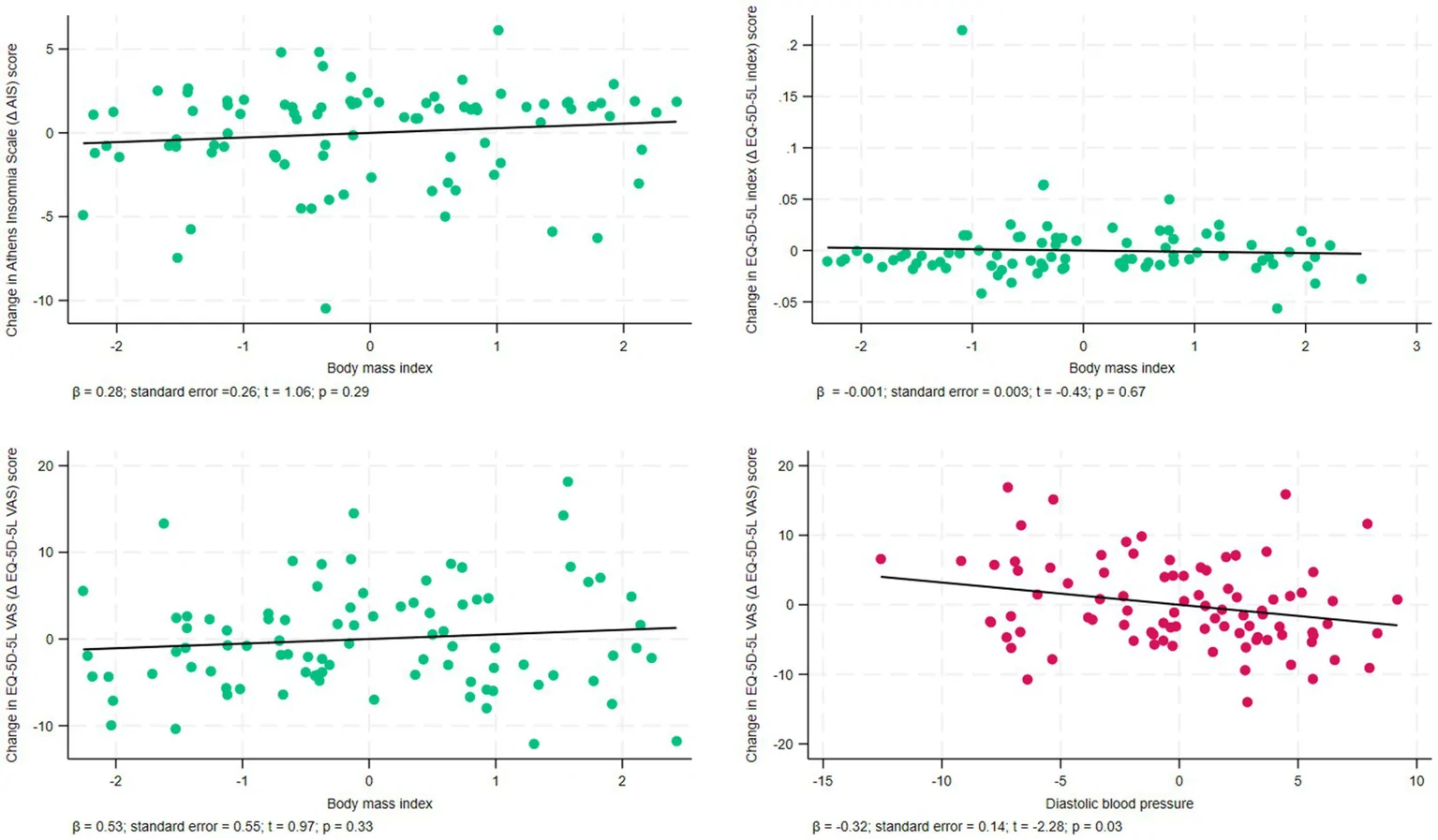
Multivariable associations between body mass index and changes in the study outcomes.

Multivariable logistic regression analysis adjusted for BMI groups [LR *χ*^2^(11) = 20.44; *p* = 0.04; pseudo *R*^2^ = 0.16] showed that classical massage (aOR = 0.17; *p* = 0.03; 95% CI: 0.034 to 0.836), mechanical massage (aOR = 0.14; *p* = 0.01; 95% CI: 0.031 to 0.650), local cryotherapy (aOR = 0.17; *p* = 0.02; 95% CI: 0.038 to 0.763), and low-level laser therapy (aOR = 0.22; *p* = 0.04; 95% CI: 0.051 to 0.908) were associated with lower odds of insomnia after comprehensive health resort treatment. Moreover, multivariable logistic regression analysis adjusted for BMI groups [LR *χ*^2^ (11) = 21.60; *p* = 0.03; pseudo *R*^2^ = 0.19] demonstrated that the application of low-level laser therapy was associated with higher odds of EQ-5D-5L index improvement (aOR = 4.47; *p* = 0.04; 95% CI: 1.06 to 18.79). Figure 7 presents coefficient plots for the administered treatment modalities and analyzed outcomes. In contrast, none of the administered modalities was significantly associated with improvement in EQ-5D-5L VAS [LR *χ*^2^ (8) = 3.08; *p* = 0.93; pseudo *R*^2^ = 0.08].

**Figure 7:**
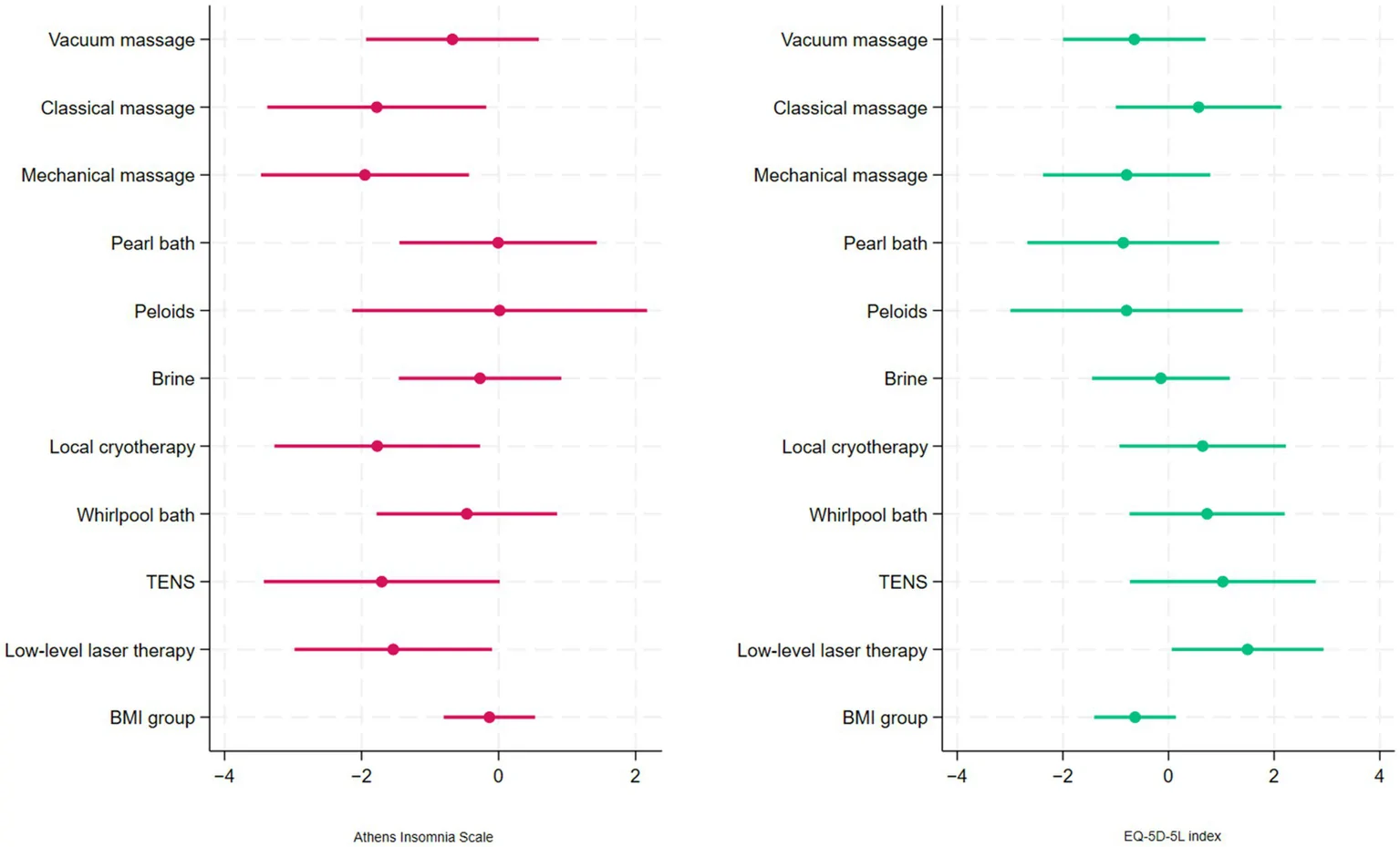
Coefficient plots of multivariable logistic regression models assessing the associations between treatment modalities and post-treatment outcomes.

Regression-based simulations showed that body weight reduction was associated with changes in ΔEQ-5D-5L VAS, whereas no association was observed for ΔAIS or ΔEQ-5D-5L index. In the weight-based model, a 3% reduction in body weight was associated with an estimated increase in ΔEQ-5D-5L VAS of 0.229 points, while in the BMI-based model the corresponding increase was 0.317 points. By contrast, the estimated effects on ΔAIS were small and statistically non-significant, and virtually no effect was observed for ΔEQ-5D-5L index. The regression coefficients for ΔEQ-5D-5L VAS were statistically significant for both body weight (*β* = −0.101; *p* = 0.043) and BMI (*β* = −0.380; *p* = 0.02). Table 6 presents the simulated associations between weight reduction and changes in patient-reported outcomes.

**Table 6:** Regression-based simulations of the association between weight reduction and changes in patient-reported outcomes.

Outcome	Predictor	*β* (95% CI)	*p*	Estimated change vs. 0% reduction at 0.5%	1.0%	1.5%	2.0%	2.5%	3.0%
ΔAIS	Weight	0.038 (−0.009 to 0.085)	0.1	−0.014	−0.029	−0.043	−0.058	−0.072	−0.087
	BMI	0.102 (−0.055 to 0.260)	0.2	−0.014	−0.029	−0.043	−0.057	−0.071	−0.086
ΔEQ-5D-5L VAS	Weight	−0.101(−0.198 to −0.003)	0.04	+0.038	+0.076	+0.114	+0.153	+0.191	+0.229
	BMI	−0.380 (−0.704 to −0.055)	0.02	+0.053	+0.106	+0.158	+0.211	+0.264	+0.317
ΔEQ-5D-5L index	Weight	0.00017 (−0.00051 to 0.00085)	0.6	−0.000	−0.000	−0.000	−0.000	−0.000	−0.000
	BMI	0.00071 (−0.00158 to 0.003)	0.5	−0.000	−0.000	−0.000	−0.000	−0.000	−0.001

β, Regression coefficient; CI, Confidence interval; *p*, *p*-value.

## 4 Discussion

The key finding of our study was that insomnia severity and quality of life improved following comprehensive health resort treatment in participants with normal weight, overweight, and class I obesity. No statistically significant differences in these improvements were detected between BMI groups. However, these findings should not be interpreted as evidence of equivalent treatment effects.

At baseline, both the prevalence and severity of insomnia were highest among participants with a BMI of 30.0–34.9 kg/m^2^ (Group 3), although the between-group differences were not statistically significant. Our results are consistent with previous reports suggesting that class I obesity is associated with sleep disturbances (4, 6). Comprehensive health resort treatment was associated with a significant reduction in the prevalence of insomnia among participants with overweight (Group 2); however, no significant differences in insomnia prevalence were observed between the BMI groups after treatment. The within-group effect sizes for the reduction in insomnia severity were moderate in participants with normal weight and class I obesity and moderate to large in those with overweight. However, the precision of these estimates varied. The confidence interval was particularly wide and close to zero in Group 1, whereas the effect was estimated more precisely in Group 2. The point estimate of the between-group effect was small however; its relatively wide 95% confidence interval indicated considerable uncertainty regarding the true magnitude of differences in AIS improvement across BMI groups. Therefore, although no statistically significant between-group differences were detected, the findings should not be interpreted as evidence of equivalent treatment effects.

Several studies have demonstrated that comprehensive health resort treatment is associated with improvements in insomnia and sleep quality. Several hypotheses may explain the treatment-mediated improvements in insomnia (19, 20, 35). During their stay at the sanatorium, participants received various therapeutic interventions, including balneotherapy, exercise therapy, and physical medicine modalities. The most frequently administered balneological treatments were peloid therapy and brine baths. According to the hypothesis proposed by Castelli et al. (19), sleep improvement may be associated with treatment-induced thermoregulatory responses, which are influenced by circadian rhythms. Moreover, exposure to balneological treatments administered at elevated temperatures increases skin temperature, which has been associated with shorter sleep onset latency. In the present study, peloids were applied at 40 °C, whereas the brine temperature ranged from 38 to 40 °C. Therefore, heat-mediated physiological mechanisms may represent one possible explanation for the observed improvements; however, their contribution was not directly assessed in the present study.

Sleep improvement may also be related to changes in pain intensity. In our study, sanatorium treatment was associated with reduced pain intensity at discharge, which may, in turn, have been related to the observed improvement in insomnia (20). Additionally, the sanatorium environment may have contributed to the observed improvement in sleep. Polish legal regulations require sanatoriums to be located in areas with at least 65% green space, while nighttime quiet hours and restrictions on environmental noise levels are mandatory (36). Available evidence suggests that exposure to green spaces is associated with improved sleep outcomes (37–39). These environmental characteristics may represent additional contextual factors that could have contributed to the observed improvements in sleep quality (40, 41).

Available reports have shown that sleep initiation is preceded by a decline in core body temperature, followed by increased peripheral blood flow and heat dissipation (42–44). Speakman (45) suggested that obesity may impair thermoregulation. Excess adipose tissue acts as a thermal insulator (46), which may reduce the efficiency of heat dissipation and thereby influence sleep onset. Although this mechanism could potentially affect sleep, it was not reflected in insomnia severity in our study. All participants took part in supervised exercise sessions, which may have enhanced thermoregulatory responses and, consequently, contributed to the alleviation of insomnia symptoms. De Graaf et al. (47) reported that the excess heat generated during exercise is dissipated through increased cutaneous vasodilation. Furthermore, regular physical exercise has been shown to stimulate nitric oxide production (48), resulting in enhanced vasodilation (49), which may also have contributed to the observed improvements in our study. Moreover, physical exercise has been shown to improve insomnia through its effects on neurotransmitter and hormone regulation, as well as mental health (50, 51). However, thermoregulatory, vascular, neurochemical, and hormonal responses were not measured in this study. Therefore, these mechanisms remain hypothetical in our participants, and we cannot determine whether exercise or other components of the comprehensive treatment acted through the pathways described above. Koçak et al. (15) demonstrated that a 3-week course of balneotherapy improved sleep quality in women with morbid obesity. Although our findings showed a similar direction of improvement across BMI groups, these results should be interpreted with caution, as participants with a BMI ≥ 35 kg/m^2^ were excluded from our study. Therefore, the absence of BMI-related differences observed in our cohort should not be extrapolated to individuals with severe obesity.

Following health resort treatment, an improvement was observed in the pain/discomfort dimension of the EQ-5D-5L, which may have contributed to the observed improvement in sleep. The therapeutic modalities administered during the treatment have previously been shown to alleviate pain through multiple mechanisms, which may have contributed to the observed improvements (20, 39, 52–55). Despite the treatment-related reduction in pain and discomfort, the remaining EQ-5D-5L dimensions did not change significantly in any BMI group. Eligibility for health resort treatment requires individuals to be independently mobile and capable of self-care (56). As the majority of participants reported no problems with mobility or self-care at admission, this may explain the absence of significant improvements in these dimensions following treatment. Symptoms of anxiety/depression were reported only in Group 2; however, no treatment-related improvement was observed in this domain. No statistically significant differences in EQ-5D-5L index values were observed between the BMI groups at either admission or discharge. The within-group effect size for improvement was moderate in Group 1, small to moderate in Group 2, and small in Group 3. The point estimate of the between-group effect was small, suggesting limited differences in improvement across BMI groups; however, the precision of this estimate should also be considered. EQ-5D-5L VAS scores improved significantly across all BMI groups, with large within-group effect sizes and relatively precise 95% confidence intervals. The between-group effect was very small, and no statistically significant differences in ΔEQ-5D-5L VAS were detected. Overall, within the BMI range examined in this study, BMI status appeared to have a limited influence on the observed improvements in self-reported health status. Nevertheless, these findings should not be interpreted as evidence of equivalent treatment effects across BMI groups.

Considering the observed improvements and the limited between-group differences, several factors may have contributed to these findings. First, participants across all BMI categories underwent a multimodal treatment program, and the combined effects of the therapeutic modalities may have contributed to the observed improvements (57). It was therefore not possible to isolate the independent causal contribution of any single component of the intervention. Although the multivariable logistic regression analyses identified associations between selected treatment modalities and lower odds of post-treatment insomnia or higher odds of improvement in the EQ-5D-5L index, these findings do not establish that the individual modalities caused the observed improvements. Because treatment components were individually selected and administered as part of a multimodal program rather than randomly assigned, these associations should be interpreted as exploratory and may reflect the combined effects of the treatment program, differences in treatment selection, or residual confounding. Furthermore, the frequency of most treatment modalities was broadly similar across BMI groups, except for vacuum massage, which was administered most frequently to participants with overweight. However, multivariable logistic regression demonstrated that vacuum massage was not significantly associated with post-treatment insomnia, EQ-5D-5L VAS, or EQ-5D-5L index outcomes. The broadly similar distribution of therapeutic modalities suggests that differences in treatment exposure were unlikely to fully explain the observed patterns. Nevertheless, the logistic regression models showed that classical and mechanical massage were associated with lower odds of insomnia after treatment. These findings are consistent with previous studies showing that various types of massage can improve sleep across a range of medical conditions (58–63). Kim et al. (64) reported that local cryotherapy did not directly improve sleep quality. However, local cryotherapy and low-level laser therapy may exert indirect beneficial effects by alleviating pain (54, 65, 66), which may, in turn, contribute to reduced insomnia symptoms. Likewise, pain reduction may partially explain the higher likelihood of obtaining better EQ-5D-5L index outcomes, as pain is a well-established determinant of health-related quality of life (67–69).

Several comorbidities that may affect general health and responses to physical medicine interventions were present in the studied population. Hypertension was the most prevalent, affecting 53.33% of participants, followed by hypothyroidism (23.33%) and diabetes mellitus (13.33%). The prevalence of these conditions did not differ significantly between BMI groups. Endothelial dysfunction and impaired vasodilatory capacity are well-recognized features of hypertension, largely resulting from the reduced bioavailability of endothelium-derived vasodilatory factors (70). Hypothyroidism has been associated with endothelial dysfunction and increased arterial stiffness (71). Diabetes mellitus is likewise associated with vascular dysfunction (72) and peripheral nerve damage (73). Peripheral artery disease was observed only in the normal-weight group and may have influenced individual responses to treatment. This condition is associated with arterial narrowing or occlusion and impaired vascular function (74, 75). Moreover, obesity is associated with reduced production and bioavailability of nitric oxide (76), a key mediator of endothelium-dependent vasodilation. These conditions and their vascular consequences could potentially modify individual responses to some components of the treatment and should therefore be considered in future studies. However, because vascular function was not directly assessed, their contribution to the observed changes in insomnia and quality of life remains uncertain.

Although some studies suggest that comprehensive balneotherapy programs may contribute to weight reduction in individuals with overweight or obesity, the available evidence remains limited, and the specific contribution of individual balneological procedures to weight loss remains unclear (14, 15, 77). In our study, improvements in insomnia and quality of life were observed in participants with normal weight, overweight, and class I obesity, with no statistically significant differences in these improvements between BMI groups. However, these findings should not be interpreted as evidence of equivalent treatment effects. Furthermore, the simulation suggested that a hypothetical 3% reduction in body weight could be related to a small improvement in self-reported health status. Given the observational nature of these analyses, these findings do not establish a causal effect of weight loss and should be confirmed in future studies specifically designed to evaluate weight-management interventions.

The study has several strengths. First, it simultaneously assessed insomnia severity and health-related quality of life before and after a complete 21-day health resort treatment program using validated instruments. Second, the comparison of participants across BMI categories enabled us to evaluate whether treatment-related changes differed according to weight status. Third, the study was conducted in a real-world clinical setting, which enhances the practical relevance of its findings. Furthermore, all individuals who initiated the treatment completed the entire program; therefore, there was no attrition due to treatment discontinuation. Finally, the multivariable analyses accounted for several clinically relevant potential confounders.

Our study has several limitations that should be acknowledged. The application of strict exclusion criteria may limit the generalizability of the findings. However, these criteria were adopted to minimize the influence of comorbidities and conditions known to be associated with sleep disturbances and impaired quality of life, thereby improving the internal validity of the study. The study sample size was not based on an *a priori* calculation, as the analysis included all eligible participants from the predefined source cohort. The relatively small sample size may have limited the statistical power to detect small between-group differences. Future studies involving larger cohorts are warranted to confirm the observed findings and enable more comprehensive subgroup analyses. Although the present study included BMI-based comparison groups, it did not include a non-treated control group. Consequently, it is not possible to determine with certainty whether the observed improvements were attributable specifically to the therapeutic intervention. Time-related changes, regression to the mean, expectancy effects, and contextual factors associated with the health resort environment may also have contributed to the observed outcomes. Additionally, the *post hoc* exclusion of participants with a BMI ≥ 35 kg/m^2^ may have introduced selection bias and may limit the generalizability of the findings. Individuals with class II or III obesity may respond differently to the treatment; therefore, the findings should be interpreted with caution and considered applicable only to individuals with normal weight, overweight, or class I obesity. Furthermore, participants undergoing health resort treatment received individually tailored therapeutic protocols based on the results of pre-treatment assessments. Although such an approach reflects routine clinical practice and supports personalized care, the lack of a standardized treatment protocol limits the ability to determine the contribution of specific therapeutic modalities to the observed outcomes. Future investigations should consider the implementation of standardized intervention protocols to enhance the comparability and scientific interpretation of the results. Additionally, BMI should be interpreted with caution, as it does not provide information regarding body composition or distinguish between fat mass and lean body mass. Future studies should incorporate more comprehensive body composition assessments, for example using bioelectrical impedance analysis performed before and after treatment completion, to better characterize the relationship between body composition and treatment effectiveness. Another limitation of the present study is that we did not assess several behavioral and socioeconomic factors that may influence sleep quality, including daytime napping, socioeconomic status, physical activity levels, and alcohol or caffeine consumption. Future studies should assess and account for these variables, as they may act as potential confounders and influence the observed treatment-related outcomes. An additional limitation is that medication use, including hypnotic, antihypertensive, antidiabetic, thyroid, and other relevant medications, was not assessed or accounted for in the analyses. Different drug classes may influence sleep quality, health-related quality of life, and responses to health resort treatment and should therefore be considered in future studies. Potential floor effects for AIS and ceiling effects for the EQ-5D-5L index may have limited the magnitude of detectable treatment-related changes. Finally, the absence of follow-up assessments represents another limitation, as it prevents evaluation of the long-term sustainability of the observed treatment-related changes.

## 5 Conclusion

Improvements in insomnia severity and quality of life were observed after comprehensive health resort treatment. Without a non-treated control group, these changes cannot be attributed with certainty to the treatment. Changes in these outcomes did not differ significantly among participants with normal weight, overweight, and class I obesity, although this finding does not establish equivalence across BMI groups. A model-based simulation suggested that a hypothetical 3% reduction in body weight might be associated with a small improvement in perceived health status. Further studies are needed to examine this simulated association.

## Data Availability

The raw data supporting the conclusions of this article will be made available by the authors, without undue reservation.
